# Deep brain stimulation (DBS) in patients with obsessive-compulsive disorder – a two-year follow-up on quality of life

**DOI:** 10.1192/j.eurpsy.2025.274

**Published:** 2025-08-26

**Authors:** V. Johansson, P. Blomstedt, M. Naesström

**Affiliations:** 1 Department of Clinical Sciences, Psychiatry unit, Umeå University, Umeå; 2 Department of Medicine Solna, Centre for Pharmacoepidemiology, Karolinska Institutet, Stockholm; 3 Department of Clinical Sciences, Neuroscience unit, Umeå University, Umeå, Sweden

## Abstract

**Introduction:**

Deep brain stimulation (DBS) is a neurosurgical procedure in which thin electrodes connected to a neuro-pacemaker are implanted into deep brain structures to modulate pathological neuronal activity with electrical current. DBS is used for symptom relief in Parkinson´s disease (Groiss *et al.* TAN Disord 2009; 2 79–91) and is under investigation for several psychiatric conditions (Naesström *et al.* NJP 2016; 70 483–91). Severe and treatment-resistant obsessive-compulsive disorder (OCD) may be treated with DBS to achieve reduced OCD symptoms, ultimately aiming for an improved quality of life.

**Objectives:**

Our objective is to present preliminary data on OCD patients’ self-reported quality of life related to the development of OCD symptoms, before DBS surgery, and up to two years after surgery.

**Methods:**

Patients with severe OCD (n=12) were enrolled in an open-label clinical trial on DBS delivered into the brain area of the bed nucleus of stria terminalis (BNST), as previously described (Naesström *et al.* W Neurosurg 2021; 149 e794-e802). The patients completed the EuroQOL five dimensions questionnaire (EQ-5D-3L) and were assessed with the Yale-Brown Obsessive Compulsive Scale (YBOCS) before DBS surgery and after six, 12, and 24 months. The paired *t-test* was used to analyze for group differences.

**Results:**

The mean age at DBS surgery was 39.2 years (standard deviation [SD] 15.5) and the baseline YBOCS score corresponded to severe to extreme OCD (mean 33, SD 3.0). The mean EQ-5D index score was 0.62 (SD 0.11) at baseline and had improved to 0.74 (SD 0.13) at the two-year follow-up and the difference was statistically significant (*t* = 2.8, *df* = 7, *p-value* = 0.025). The EQ-5D VAS scores measured pre-surgery were low (mean 35.9, SD 22.2) and had increased two years post-surgery (mean 54.4, SD 21.0), but with no statistically significant difference (*t* = 1.9, *df* = 8, *p-value* = 0.093).

**Conclusions:**

Quality of life in OCD patients two years after DBS surgery measured with the EQ-5D-3L showed an improvement two years following surgery for the EQ-5D index but not for the VAS scale. These preliminary data show that self-assessment with the EQ-5D-3L scale may be used to follow up on patients’ quality of life after DBS and longer follow-up periods are warranted.

**Disclosure of Interest:**

None Declared

